# Oral Administration of Astrovirus Capsid Protein Is Sufficient To Induce Acute Diarrhea *In Vivo*

**DOI:** 10.1128/mBio.01494-16

**Published:** 2016-11-01

**Authors:** Victoria A. Meliopoulos, Shauna A. Marvin, Pamela Freiden, Lindsey A. Moser, Prashant Nighot, Rizwana Ali, Anthony Blikslager, Muralidhar Reddivari, Richard J. Heath, Matthew D. Koci, Stacey Schultz-Cherry

**Affiliations:** a Department of Infectious Diseases, St. Jude Children’s Research Hospital, Memphis, Tennessee, USA; b Department of Pathobiological Sciences, School of Veterinary Medicine, University of Wisconsin—Madison, Madison, Wisconsin, USA; c Department of Clinical Sciences, North Carolina State University, Raleigh, North Carolina, USA; d Department of Poultry Science, North Carolina State University, Raleigh, North Carolina, USA

## Abstract

The disease mechanisms associated with the onset of astrovirus diarrhea are unknown. Unlike other enteric virus infections, astrovirus infection is not associated with an inflammatory response or cellular damage. *In vitro* studies in differentiated Caco-2 cells demonstrated that human astrovirus serotype 1 (HAstV-1) capsid protein alone disrupts the actin cytoskeleton and tight junction complex, leading to increased epithelial barrier permeability. In this study, we show that oral administration of purified recombinant turkey astrovirus 2 (TAstV-2) capsid protein results in acute diarrhea in a dose- and time-dependent manner in turkey poults. Similarly to that induced by infectious virus, TAstV-2 capsid-induced diarrhea was independent of inflammation or histological changes but was associated with increased intestinal barrier permeability, as well as redistribution of sodium hydrogen exchanger 3 (NHE3) from the membrane to the cytoplasm of the intestinal epithelium. Unlike other viral enterotoxins that have been identified, astrovirus capsid induces diarrhea after oral administration, reproducing the natural route of infection and demonstrating that ingestion of intact noninfectious capsid protein may be sufficient to provoke acute diarrhea. Based on these data, we hypothesize that the astrovirus capsid acts like an enterotoxin and induces intestinal epithelial barrier dysfunction.

## IMPORTANCE

Acute gastroenteritis, with its sequela diarrhea, is one of the most important causes of childhood morbidity and mortality worldwide. A variety of infectious agents cause gastroenteritis, and in many cases, an enterotoxin produced by the agent is involved in disease manifestations. Although we commonly think of bacteria as a source of toxins, at least one enteric virus, rotavirus, produces a protein with enterotoxigenic activity during viral replication. In these studies, we demonstrate that oral administration of the turkey astrovirus 2 (TAstV-2) structural (capsid) protein induces acute diarrhea, increases barrier permeability, and causes relocalization of NHE3 in the small intestine, suggesting that rotavirus may not be alone in possessing enterotoxigenic activity.

## OBSERVATION

Diarrhea is one of the most common causes of childhood morbidity and mortality worldwide, causing 2 million deaths and 1.4 billion nonfatal cases each year (1). In the United States, 220,000 children younger than 5 years of age are hospitalized each year with gastroenteritis, accounting for approximately 9% of all hospitalizations in this age group (2).

Astroviruses are leading causes of gastroenteritis in children under 2 years of age, people who are immunocompromised, and older adults (3, 4). They are small, nonenveloped viruses belonging to the family *Astroviridae* that possess single-stranded, positive-sense RNA genomes with three open reading frames (ORFs): ORF1a, ORF1b, and ORF2 (5). These encode the viral nonstructural proteins, the viral RNA-dependent RNA polymerase, and the viral capsid protein, respectively. Astroviruses have been isolated from the young of a variety of species, including humans, and are typically associated with an acute self-limiting diarrhea (5, 6). Infection with human astrovirus serotype 1 (HAstV-1) is most commonly detected, though 8 canonical serotypes of HAstV (HAstV-1 to -8) and several noncanonical human genogroups have been isolated with various frequencies (3–6).

Despite the prevalence of astrovirus-induced diarrhea, little is known about the mechanism by which astroviruses cause diarrhea, primarily due to the lack of animal models. *In vivo* studies in turkey astrovirus 2 (TAstV-2)-infected turkey poults (currently the best-characterized small-animal model for gastroenteritis) demonstrated only mild histological changes in the absence of intestinal lesions, cell death, or inflammation (4, 7, 8). Similar observations were made in an astrovirus-infected child (9). Using differentiated Caco-2 intestinal epithelial cells *in vitro*, we demonstrated that astrovirus infection led to increased barrier permeability through disruption of the actin cytoskeleton and reorganization of the tight junction complex (10). The astrovirus-mediated increase in barrier permeability was independent of productive viral replication; UV-inactivated virus and purified recombinant HAstV-1 capsid protein alone both increased barrier permeability. The goal of these studies was to extend our *in vitro* findings and determine if oral administration of the TAstV-2 capsid protein induced diarrhea and caused dysregulation of intestinal permeability.

Here, we demonstrate that the astrovirus capsid protein alone is sufficient to induce acute diarrhea in a small-animal model (turkey poults) in a dose-dependent manner. Consistent with our *in vitro* results (10), purified recombinant capsid protein induced a disruption of intestinal barrier function, as defined by a drop in transepithelial electrical resistance (TER) and increased mannitol permeability in the small intestine. Digestion of the capsid with proteinase K caused a loss of activity, suggesting that the capsid structure, or at least more than a short peptide sequence, is required for induction of diarrhea. Capsid administration also resulted in a redistribution of sodium hydrogen exchanger 3 (NHE3) from the membrane to the cytoplasm of the intestinal epithelium, similar to that observed in TAstV-2 infection (8, 11). Our data suggest that the astrovirus capsid protein can act as a viral enterotoxin by increasing intestinal permeability and possibly impairing sodium absorption, a novel mechanism of action for a viral capsid protein. However, other mechanisms, including regulation of anion secretion, warrant further investigation.

### Oral administration of TASTV-2 capsid protein induces diarrhea *in vivo*.

**FIG 1:**
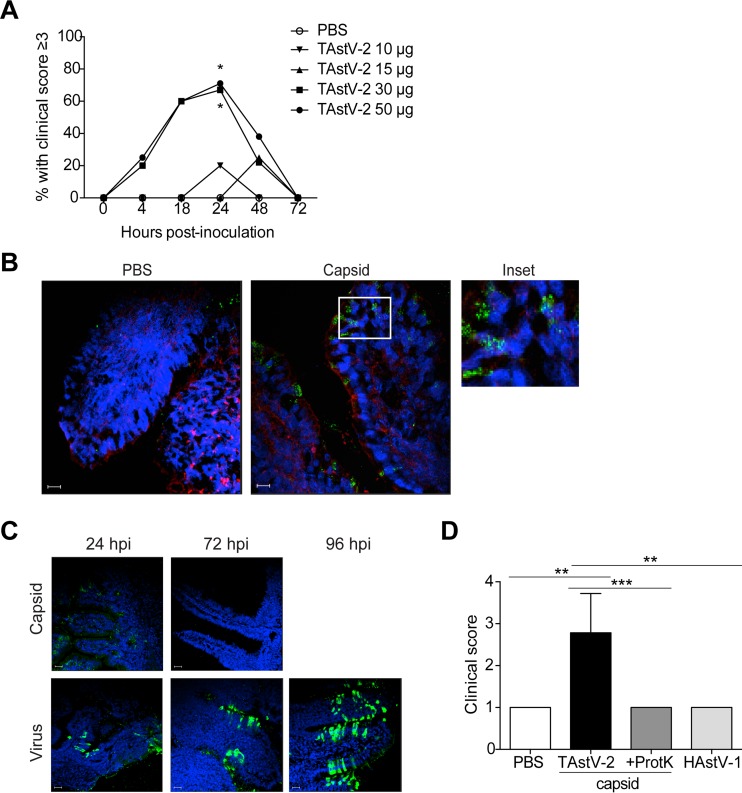
TAstV-2 capsid protein induces time- and dose-dependent diarrhea. Turkey poults (*n* = 5 to 9 per group) were orally inoculated with PBS alone or 50 µg of purified recombinant TAstV-2 capsid in PBS. (A) Percentage of TAstV-2 capsid-treated animals with a clinical score of 3 or higher over time. (B) At 6 h postinoculation, poults were sacrificed and intestinal sections were stained for TAstV-2 capsid (green), actin (red), and nuclei (blue) and examined by immunofluorescence microscopy. Bar, 20 µm. (C) TAstV-2 capsid and virally inoculated poults were sacrificed at the indicated time points, and intestinal sections were stained for TAstV-2 capsid (green) and nuclei (blue) and examined by immunofluorescence assay. Bar, 20 µm. (D) Turkey poults (*n* = 3 to 7 per group) were inoculated with PBS, 50 µg TAstV-2 capsid, 50 µg of proteinase K (ProtK)-digested TAstV-2 capsid, or 50 µg HAstV-1 capsid and scored for diarrhea at 24 h posttreatment. *, *P* < 0.05; **, *P* < 0.01; ***, *P* < 0.001.

To determine if the TAstV-2 capsid protein was sufficient to cause diarrhea *in vivo*, 5-day-old commercial turkey poults were orally inoculated with endotoxin-free purified recombinant TAstV-2 capsid protein (0 to 50 µg per poult) in phosphate-buffered saline (PBS). The inoculated poults were individually monitored for diarrhea by using a clinical scoring chart modified from that of Ball et al. (12) (see Fig. S1A in the supplemental material). We considered a score of 3 or higher to indicate diarrhea. Oral administration of TAstV-2 capsid resulted in an increased clinical score in a time- and dose-dependent manner (Fig. 1A). PBS-inoculated poults did not develop diarrhea, and animals receiving 10 or 15 µg of TAstV-2 capsid rarely scored higher than PBS-inoculated poults (Fig. 1A). In contrast, poults administered the highest concentrations of capsid, 30 and 50 µg, had elevated scores within 4 h postinoculation (hpi) that continued to increase through 24 hpi, at which time 75% of the poults were symptomatic. Clinical scores decreased by 48 hpi, and poults had completely recovered by 72 hpi (Fig. 1A). Poults receiving the highest capsid concentrations had increased volumes and frequencies of stool, and poults were covered in brown, pasty feathers, indicating extensive fecal incontinence. Immunofluorescent microscopic examination of intestinal sections collected 6 hpi confirmed that the TAstV-2 capsid protein was localized to the apical membrane of the enterocytes (Fig. 1B), similarly to virus localization in TAstV-2-infected poults (7). These results demonstrate that TAstV-2 capsid protein was able to reach the gut and induce diarrhea via the natural route of infection in a time- and dose-dependent manner. An important question is whether we are administering a biologically relevant capsid concentration. To calculate this, we used the molecular weight of the capsid protein and the knowledge that the virus particle is comprised of 180 molecules of capsid protein (13) to estimate that 50 μg of TAstV-2 capsid protein is roughly equivalent to 2 × 10^12^ particles (R. M. DuBois, personal communication). Inoculating poults with 10^13^ genomic units of TAstV-2 did not result in clinical disease until 3 days postinoculation (dpi) (7) (see Fig. S1B in the supplemental material). Thus, we monitored the TAstV-2 genomic units in stool at 6, 24, 48, 72, and 96 hpi and intestinal homogenates at 24 and 72 hpi by quantitative real-time reverse transcription-PCR (RT-PCR). TAstV-2 genomic levels in stool were 10^10^ by 6 hpi and did not reach levels greater than 2 × 10^12^ copies per gram of stool until 3 dpi, the point at which clinical signs are observed (see Fig. S1C in the supplemental material). A similar trend was seen in the intestines, where copy number was less than 2 × 10^12^ per µg of intestinal homogenate at 24 hpi but reached 2 × 10^13^ copies at 72 hpi (see Fig. S1D in the supplemental material).

We took several approaches to translate this to actual capsid levels during infection. First, we performed a capsid-specific enzyme-linked immunosorbent assay (ELISA) on the stool and intestinal homogenates described above. Unfortunately, the presence of inhibitors in the homogenates made it impossible to detect low protein levels. This is highlighted by the dramatic decrease in absorbance when a known concentration of capsid was diluted in homogenate instead of PBS (see Fig. S2 in the supplemental material). Our attempts to develop a capture ELISA using available antibodies were unsuccessful. Next, we performed immunofluorescence microscopy for capsid protein on capsid- or TAstV-2-inoculated intestines at 24, 72, and 96 (virus only) hpi. There was a marked increase in capsid staining over time in TAstV-2-inoculated poults, while capsid levels decreased in the poults administered recombinant capsid (Fig. 1C). As expected, the staining patterns differed between virus- and capsid-treated poults, making it difficult to directly compare or quantitate the fluorescence, yet it is evident that capsid levels in the TAstV-2-inoculated poults were much higher than poults administered recombinant capsid. Taken together, these studies suggest that the doses of capsid used are likely achieved during infection, highlighting the novelty of our finding: orally administering a biologically relevant concentration of TAstV-2 capsid protein results in acute diarrhea.

To determine if intact capsid protein was required for activity, TAstV-2 capsid protein was digested with proteinase K overnight and dialyzed against PBS, and 50 µg of digested capsid protein was orally administered to poults. At 24 hpi, animals receiving proteinase K-digested TAstV-2 capsid had clinical scores similar to those of PBS-treated controls, whereas animals receiving intact TAstV-2 capsid had significantly higher scores (Fig. 1D). Treatment with proteinase K resulted in complete digestion of the capsid, as evidenced by PageBlue protein staining (see Fig. S3A in the supplemental material). The ~30-kDa band in lane 2 of Fig. S3A in the supplemental material is likely proteinase K, which is 28.9 kDa, given that Western blot analysis using 3 distinct polyclonal antibodies to known TAstV-2 capsid epitopes did not detect capsid protein (see Fig. S3B in the supplemental material). Finally, to determine if capsid-induced diarrhea was specific to the TAstV-2 capsid, poults were inoculated with 50 µg of purified recombinant HAstV-1 capsid protein. No increase in clinical score was noted (Fig. 1D), suggesting that capsid-associated diarrhea is specific to TAstV-2 capsid and that the intact protein, or at least more than a small peptide sequence, is required for induction of diarrhea by the capsid *in vivo*.

### TASTV-2 capsid increases barrier permeability.

**FIG 2:**
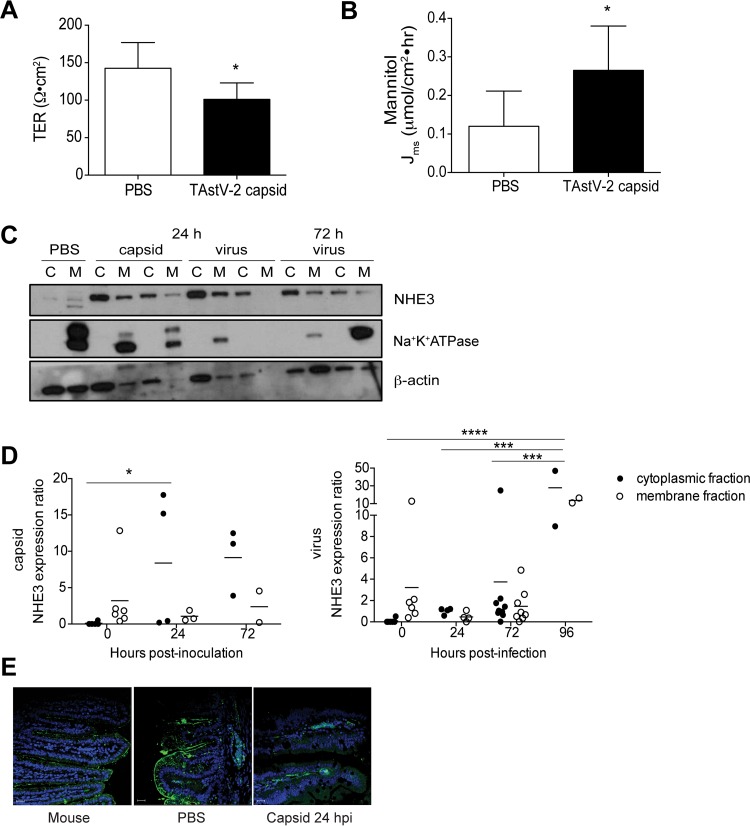
Administration of TAstV-2 capsid induces *in vivo* barrier permeability and disrupts NHE3 localization. (A and B) At 6 h postinoculation (hpi), intestines (*n* = 6) were harvested from treatment and control groups and mounted in Ussing chambers. Transepithelial electrical resistance (TER) (A) and mucosal-to-serosal flux of [^3^H]mannitol (B) were assayed as detailed in Text S1 in the supplemental material. (C) Turkey poults were inoculated with capsid protein or TAstV-2. At the indicated times, intestines were isolated and separated into membrane (M) or cytosolic (C) fractions. Proteins (5 µg) were separated by SDS-PAGE and probed for NHE3, Na^+^K^+^-ATPase, or β-actin. Samples were run in duplicate. (D) NHE3 localized in each fraction was quantified using ImageJ from six independent blots*.* NHE3 expression was normalized to β-actin. Na^+^K^+^-ATPase expression was used as a fractionation control. (E) PBS- and TAstV-2 capsid-inoculated turkey poults (*n* = 2 to 4 per group) were sacrificed at 24 hpi, and intestinal sections were stained for SGLT-1 (green) and nuclei (blue) and examined by immunofluorescence microscopy. Bar, 20 µm. Asterisks represent statistically significant differences compared to controls. *, *P* < 0.05; ***, *P* < 0.001; ****, *P* < 0.0001.

We demonstrated that the HAstV-1 capsid protein increased barrier permeability in differentiated Caco-2 cells (10) and hypothesized that this was a mechanism for astrovirus induction of diarrhea. To determine if TAstV-2 capsid protein increased intestinal barrier permeability *in vivo*, sections of small intestine were collected from poults inoculated with PBS (control) or 50 µg TAstV-2 capsid at 6 hpi (*n* = 6), mounted in Ussing chambers, and monitored for changes in transepithelial resistance (TER) and mannitol flux, which are measurements of barrier permeability (14). TAstV-2 capsid protein markedly reduced intestinal barrier function as evidenced by decreased TER and increased mannitol flux, indicative of increased paracellular permeability (Fig. 2A and B). The increased barrier permeability occurred in the absence of cellular damage or an inflammatory response (see Fig. S4 in the supplemental material), similarly to our previous findings with TAstV-2 infection (7). To determine if there was reorganization of tight junction proteins similar to what we observed *in vitro*, intestinal sections were stained for a variety of cellular junction proteins using murine intestines as a positive control. Unfortunately, none of the commercially available antibodies recognized turkey occludin, ZO-1, claudin 1, claudin 3, or connexin (see Fig. S5 in the supplemental material). Regardless, Ussing chamber studies clearly demonstrate that oral administration of the TAstV-2 capsid results in increased barrier permeability. Multiple studies have demonstrated that increased barrier permeability may be insufficient to cause diarrhea (15–18). Specifically, Clayburgh et al. showed that both barrier dysfunction and inhibition of the epithelial brush border sodium hydrogen exchanger 3 (NHE3) were required for tumor necrosis factor alpha (TNF-α)-mediated diarrhea *in vivo* (15). TAstV-2 infection alters the localization of NHE3 from the membrane to the cytosol in intestinal segments, leading to sodium malabsorption and potentially inducing an osmotic diarrhea (8). Thus, we assessed not only the localization of NHE3 in capsid and TAstV-2 inoculated poults but also the sodium-glucose transporter 1 (SGLT-1). It has been previously reported that rotavirus induces diarrhea in part by inhibiting SGLT-1 activity without altering overall protein levels (19). Sections of small intestine were collected at different times, lysed, and separated into membrane and cytoplasmic fractions to monitor NHE3 localization. The membrane marker Na^+^K^+^-ATPase was used as a fractionation control. In PBS-inoculated poults, NHE3 was primarily located in the membrane fraction (Fig. 2C and D [quantification]). In contrast, NHE3 was significantly increased in the cytoplasmic fractions of capsid-inoculated poults at 24 hpi (Fig. 2D), similarly to that observed in TAstV-2-infected poults (8). Unfortunately, the SGLT-1 antibody did not recognize turkey SGLT-1 by Western blotting but did work by immunofluorescence microscopy, where we observed a reorganization in capsid-inoculated intestines at 24 hpi (Fig. 2E). Overall, these studies suggest that astrovirus-induced diarrhea may involve mislocalization of transporter proteins in addition to increasing barrier permeability, both of which can be induced by the capsid protein alone. Ongoing work is needed to investigate other mechanisms of action, including modulation of anion secretion by the virus and/or capsid.

### Conclusions.

Our studies suggest that oral administration (i.e., the natural route of infection) of the TAstV-2 capsid protein induces clinical disease. Diarrheal onset is accompanied by increased permeability and redistribution of two sodium transporters, NHE3 and SGLT-1. Unlike TAstV-2 viral infection, where diarrhea continues for at least 12 dpi (7, 8), capsid-induced disease is acute, waning by 72 hpi, and is dose dependent. Doses of less than 30 μg cause little if any clinical disease in the majority of poults. The acute and transient nature of the capsid-induced clinical disease is expected, as progeny capsid particles are not produced in the absence of productive infection; there cannot be amplification or extension of disease as observed during a typical infection. Induction of clinical disease appears to be specific to the TAstV-2 capsid, given that administration of digested capsid or recombinant human HAstV-1 capsid, which increases barrier permeability in Caco-2 cells (10), has no effect. One possible explanation is that HAstV-1 capsid fails to bind to turkey enterocytes or that postentry events differ between TAstV-2 and HAstV-1. The exact mechanism(s) by which astroviruses cause diarrhea remains unknown, and we cannot rule out the contribution of other astrovirus proteins during infection. We previously demonstrated that TAstV-2-induced disease was independent of cellular damage or induction of an inflammatory response (7) but likely involves sodium malabsorption (8). Our *in vitro* studies demonstrated that the HAstV-1 capsid protein alone disrupted tight junction complexes, leading to increased barrier permeability (10). Thus, we hypothesized that TAstV-2-induced diarrhea resulted, at least in part, from the capsid protein binding to enterocytes and inducing changes in barrier permeability. Oral administration of TAstV-2 capsid protein led to its localization to the apical membranes of enterocytes, a marked decrease in TER, and an increase in mannitol flux as an indicator of increased paracellular permeability by 6 hpi; these data are consistent with the ability of purified recombinant HAstV-1 capsid protein to reorganize the tight junction complex and increase barrier permeability in differentiated Caco-2 intestinal epithelial cells (10). Unfortunately, none of the commercial antibodies recognized turkey junctional proteins, so we were unable to demonstrate reorganization of specific proteins, although previous studies describe a role for occludin and F-actin reorganization as a consequence of HAstV-1 infection (7, 10). Regardless, increased permeability alone may be insufficient to cause diarrhea. However, when combined with relocalization of sodium transporters away from the plasma membrane, disruption of fluid and electrolyte transport leads to a net loss of liquid in the intestine and subsequently diarrhea (17). Decreasing the apical membrane levels of NHE3 can lessen its activity, in turn reducing Na^+^ absorption (20). We hypothesize that both a decrease in epithelial permeability and inhibition of sodium transport proteins are required for induction of diarrhea by the TAstV-2 capsid. Future studies are required to understand the interplay, if any, between the regulation of tight junctions and the actin cytoskeleton through receptor binding and the reorganization of sodium and possibly other transporters that leads to malabsorption. The consistency between the increase in intestinal barrier permeability *in vitro* caused by HAstV-1 and that *in vivo* caused by TAstV-2 supports a general model of astrovirus capsid-mediated disruption of intestinal permeability, potentially leading to induction of transient diarrhea. However, other causative mechanisms for diarrhea, such as anion secretion, warrant further investigation.

We used multiple methods to determine if we were administering a biologically relevant capsid concentration. These studies were complicated by the lack of cell culture systems for TAstV-2 and insufficient tools to develop a capture ELISA. Regardless, based on our calculations, development of a quantitative real-time RT-PCR assay, and microscopic detection of capsid levels in the intestines, our studies suggest that 30- to 50-µg levels of capsid are reached at 3 dpi during a TAstV-2 infection, when diarrhea begins.

Enterotoxins are defined as macroproteins that act in the intestine during infection and, in most cases, induce diarrhea (21). Our data suggest that the TAstV-2 capsid could be considered a viral enterotoxin. Our studies uniquely demonstrate disease when the enterotoxin is administered via oral ingestion, the natural route of infection. Work with rotavirus NSP4 protein, the first identified viral enterotoxin, required intraperitoneal or intraileal administration to ileal loop sections to induce diarrhea (12, 22). The NSP4 protein and specific NSP4 peptides induce diarrhea in neonatal mice in the absence of histological damage to the intestinal mucosa (23) through a number of mechanisms, including impairment of glucose absorption and increasing chloride reabsorption (reviewed in reference 19). The simian immunodeficiency virus (SIV) envelope glycoprotein may also contain an enterotoxin domain, as surgical introduction of the surface unit envelope glycoprotein into mouse intestinal loops resulted in fluid accumulation in the absence of cellular damage (22). Our findings raise an interesting question as to whether ingestion of even noninfectious astrovirus particles could result in acute diarrhea. However, further studies and development of mammalian models to study astrovirus pathogenesis, particularly human astrovirus genotypes, are required to fully understand the impact of exposure to noninfectious particles, particularly in food and/or water sources.

## SUPPLEMENTAL MATERIAL

Figure S1  TAstV-2 infection in turkey poults. (A) Clinical scoring chart to assign diarrhea scores. (B) Turkey poults (*n* = 4 to 6 per group) were orally inoculated with 1.5 × 10^13^ genome copies of TAstV-2 and were monitored for clinical score reported as the percentage of animals with diarrhea (score of 3 or higher) over time. (C and D) Viral copy number was monitored on stool (C) and intestinal homogenates (D) by quantitative real-time RT-PCR at the indicated times. Download Figure S1, TIF file, 2.2 MB

Figure S2  Quantification of TAstV-2. (A) ELISA was performed on TAstV-2 capsid (25 ng) serially diluted 2-fold in PBS or intestinal homogenate from uninfected poults (control), viral homogenate, or control homogenate alone. Download Figure S2, TIF file, 0.9 MB

Figure S3  Proteinase K digestion of TAstV-2 capsid. (A) PageBlue protein staining of whole and proteinase K-digested TAstV-2 capsid (50 µg). (B) Purified TAstV-2 capsid was incubated with proteinase K overnight at 37°C at a final concentration of 50 µg/ml and then treated with protease inhibitor cocktail and dialyzed using a 7-kDa-molecular-mass cutoff microdialysis cassette in PBS. Capsid degradation was confirmed by immunoblotting assay using anti-TAstV-2 capsid antibodies. Download Figure S3, TIF file, 0.4 MB

Figure S4  TAstV-2 capsid inoculation does not cause intestinal damage or inflammation. Intestinal sections collected from turkey poults inoculated orally with PBS (control) or 50 µg recombinant TAstV-2 capsid protein (capsid) were collected at 6 hpi and stained with hematoxylin and eosin. Bar, 25 µm. Download Figure S4, TIF file, 0.5 MB

Figure S5  Immunofluorescent staining of turkey intestines for tight junction proteins. Uninfected mice or turkey poults were sacrificed, and intestinal sections were stained for tight junction proteins (green) and nuclei (blue). Bar, 20 µm. Download Figure S5, TIF file, 1.7 MB

Text S1  Supplemental methods. Download Text S1, DOCX file, 0.1 MB
