# Supplementary figures and images for: Oral Administration of Astrovirus Capsid Protein Is Sufficient To Induce Acute Diarrhea In Vivo
Source: mBio. 2016 Nov 1;7(6):e01494-16. doi: 10.1128/mBio.01494-16 (PMC5090040; doi:10.1128/mBio.01494-16)

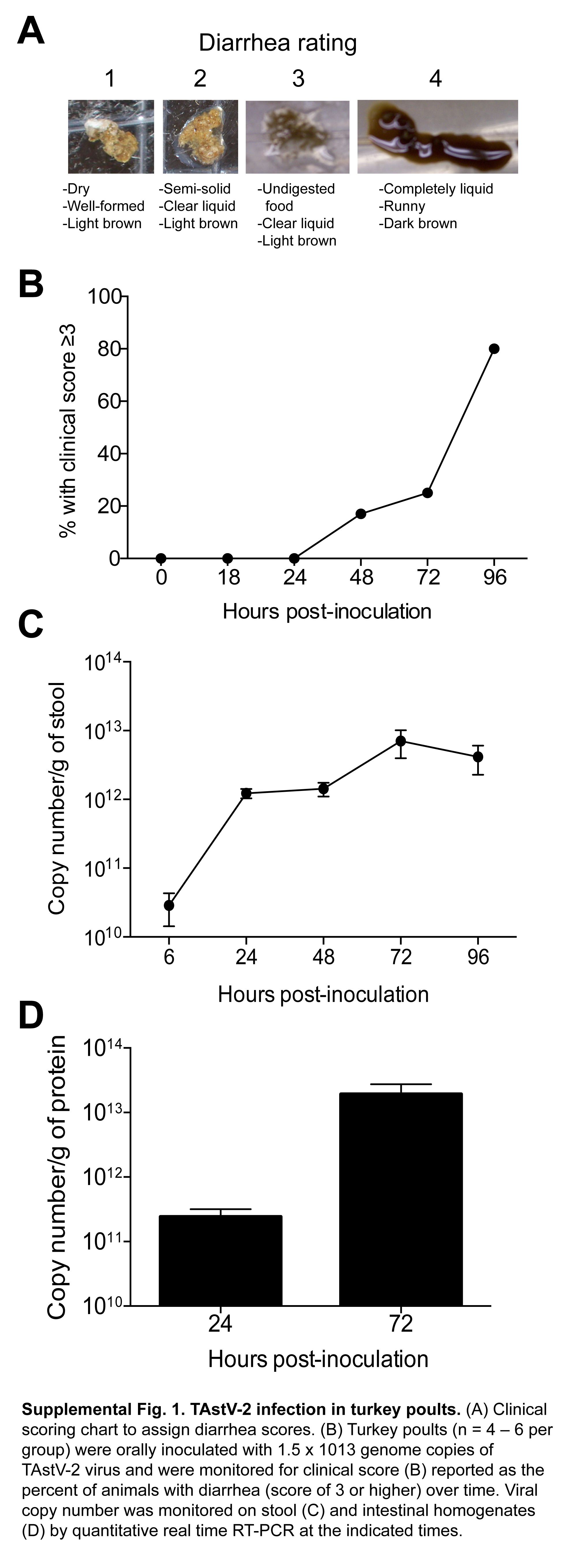

Supplement: Figure S1 — TAstV-2 infection in turkey poults. (A) Clinical scoring chart to assign diarrhea scores. (B) Turkey poults (n = 4 to 6 per group) were orally inoculated with 1.5 × 10^13^ genome copies of TAstV-2 and were monitored for clinical score reported as the percentage of animals with diarrhea (score of 3 or higher) over time. (C and D) Viral copy number was monitored on stool (C) and intestinal homogenates (D) by quantitative real-time RT-PCR at the indicated times. Download [file mbo005163046sf1.tif]

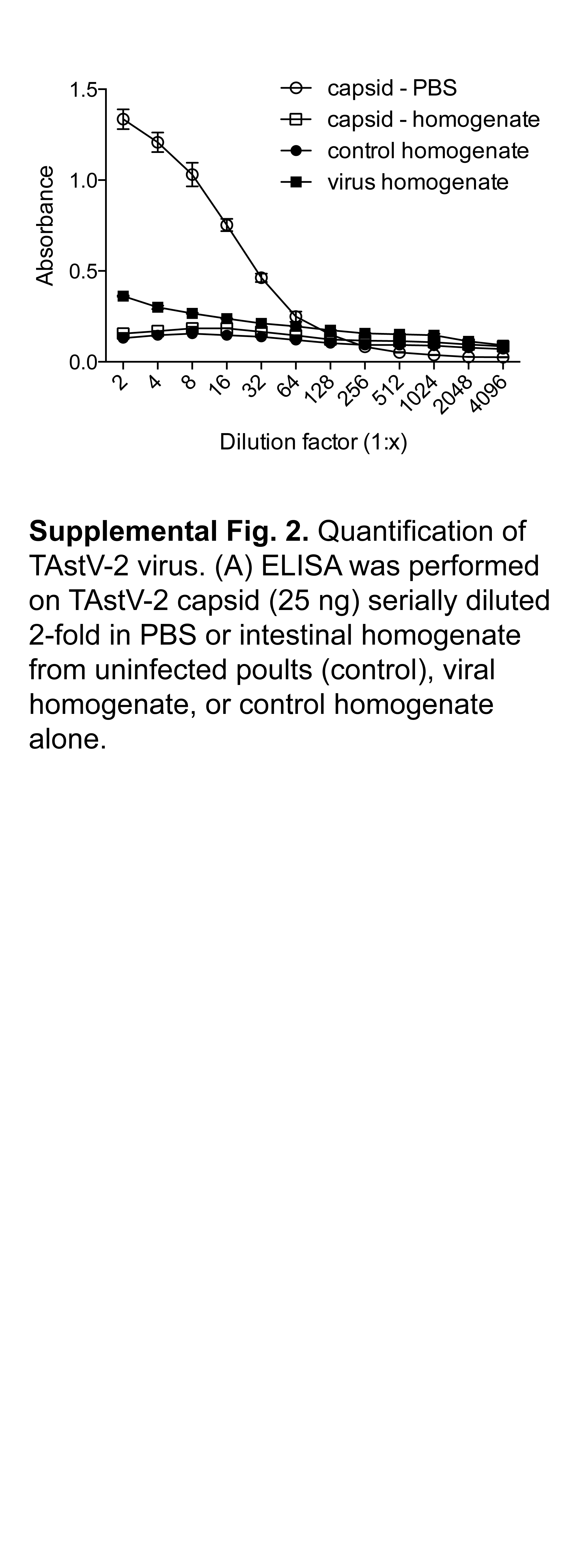

Supplement: Figure S2 — Quantification of TAstV-2. (A) ELISA was performed on TAstV-2 capsid (25 ng) serially diluted 2-fold in PBS or intestinal homogenate from uninfected poults (control), viral homogenate, or control homogenate alone. Download [file mbo005163046sf2.tif]

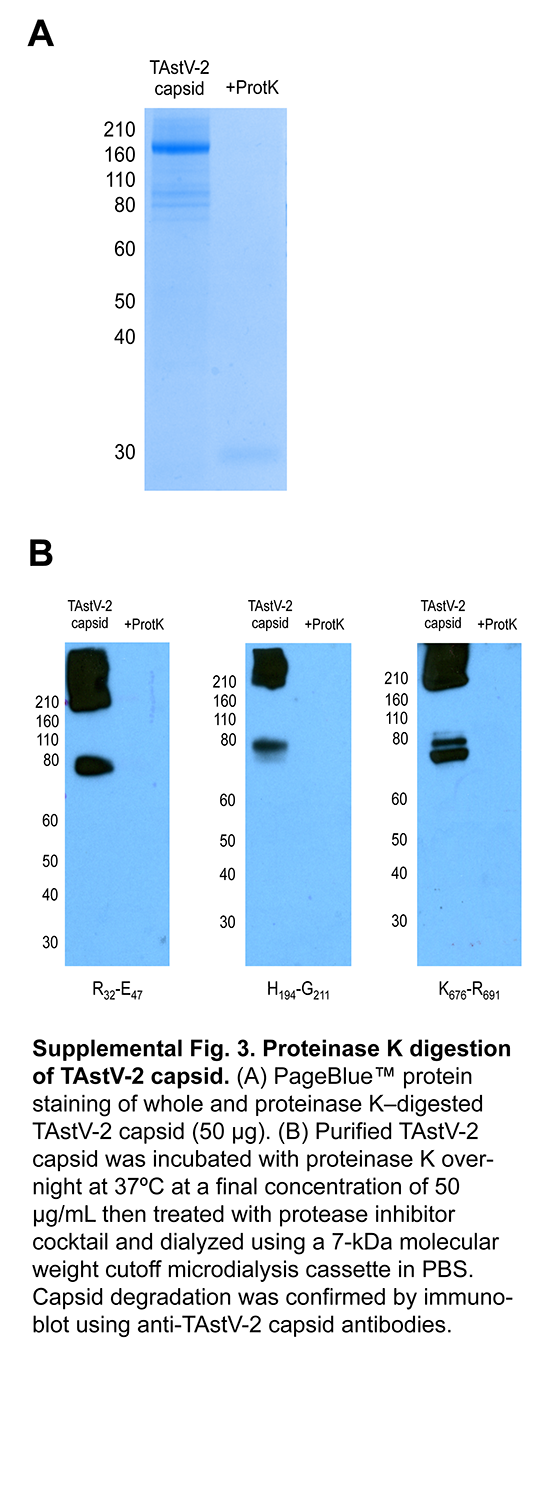

Supplement: Figure S3 — Proteinase K digestion of TAstV-2 capsid. (A) PageBlue protein staining of whole and proteinase K-digested TAstV-2 capsid (50 µg). (B) Purified TAstV-2 capsid was incubated with proteinase K overnight at 37°C at a final concentration of 50 µg/ml and then treated with protease inhibitor cocktail and dialyzed using a 7-kDa-molecular-mass cutoff microdialysis cassette in PBS. Capsid degradation was confirmed by immunoblotting assay using anti-TAstV-2 capsid antibodies. Download [file mbo005163046sf3.tif]

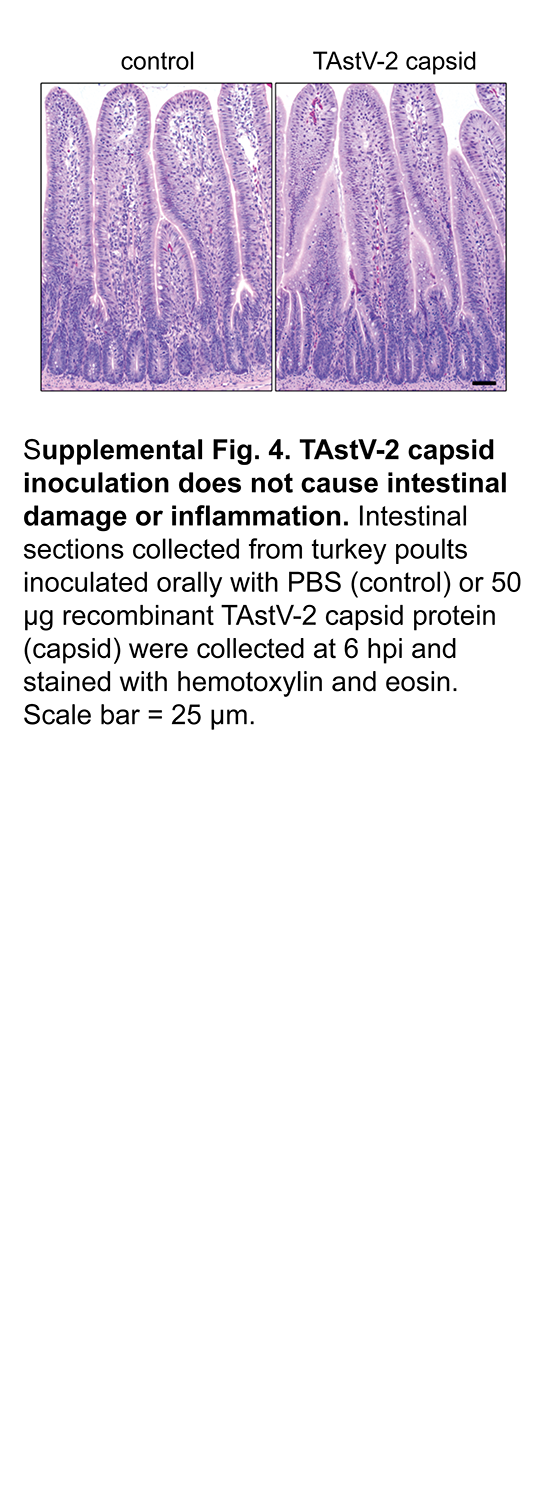

Supplement: Figure S4 — TAstV-2 capsid inoculation does not cause intestinal damage or inflammation. Intestinal sections collected from turkey poults inoculated orally with PBS (control) or 50 µg recombinant TAstV-2 capsid protein (capsid) were collected at 6 hpi and stained with hematoxylin and eosin. Bar, 25 µm. Download [file mbo005163046sf4.tif]

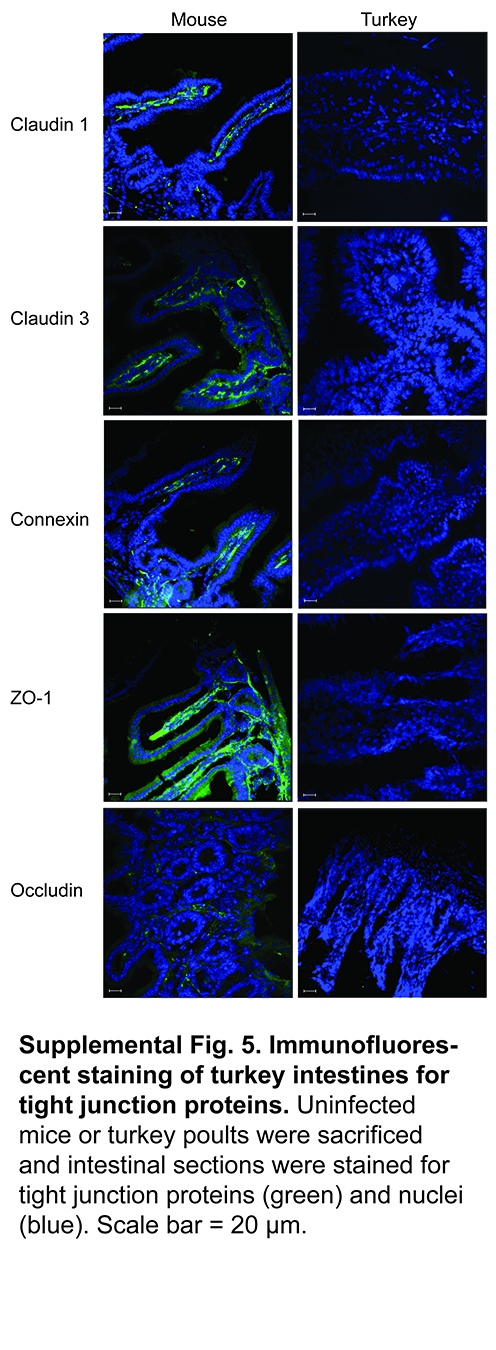

Supplement: Figure S5 — Immunofluorescent staining of turkey intestines for tight junction proteins. Uninfected mice or turkey poults were sacrificed, and intestinal sections were stained for tight junction proteins (green) and nuclei (blue). Bar, 20 µm. Download [file mbo005163046sf5.tif]
