# Supplementary material for: Oral Administration of Astrovirus Capsid Protein Is Sufficient To Induce Acute Diarrhea In Vivo
Source: mBio. 2016 Nov 1;7(6):e01494-16. doi: 10.1128/mBio.01494-16 (PMC5090040; doi:10.1128/mBio.01494-16)
Supplement: Text S1 — Supplemental methods. Download [file mbo005163046s1.docx]

**Supplemental Materials Text S1**

**Animal ethics statement**

All animal work was approved by the Institutional Animal Care and Use Committees of St. Jude Children’s Research Hospital and North Carolina State University (NCSU) in accordance with guidelines established by the Institute of Laboratory Animal Resources and approved by the Governing Board of the U.S. National Research Council. Both St. Jude Children’s Research Hospital and NCSU are fully accredited by the Association for the Assessment and Accreditation of Laboratory Animal Care International (AAALAC-I) and have approved Animal Welfare Assurance Statements on file with the Office of Laboratory Animal Welfare (A3077-01 and A3331-01 respectively).

**Purification of recombinant capsid proteins**

The TAstV-2 and HAstV-1 ORF2 genes, which encode the full-length capsid protein, were cloned into the baculovirus transfer vectors pFastBacHT-A and 1392, respectively, and were produced by the St. Jude Children’s Research Hospital Protein Production Core Facility as described ([1-3](#_ENREF_1)). Briefly, bacmid production, transfection, and baculovirus amplification were carried out according to the manufacturer’s instructions (Invitrogen , Grand Island, NY). Suspension cultures of *Sf*9 insect cells (ATCC, Manassas, VA) were grown in SFX-insect serum-free media to a density of 2 × 10^6^ cells/mL and infected at a multiplicity of infection of 10. Cultures were gently shaken for 3 days at 28°C, then cells were collected by centrifugation (2400 x *g*) and resuspended in 50 mM Tris, pH 7.9, 500 mM NaCl, and 10% glycerol buffer. Cell pellets were then lysed by microfluidization and clarified by centrifugation at 48,000 x *g*. Soluble TAstV-2 and HAstV-1 capsids were purified from the supernatant by HisTrap metal affinity chromatography. Purified capsids were dialyzed into PBS, pH 7.6, flash frozen in liquid nitrogen, and stored indefinitely at −80°C. Endotoxin levels were less than 0.2 EU/mL, as determined by the Limulus Amebocyte Lysate Assay (Lonza, Walkersville, MD). Protein concentrations were determined by BCA assay (Pierce, Rockford, IL), and purification was confirmed by Western blot analysis.

**Proteinase K digestion**

Purified TAstV-2 capsid was incubated with proteinase K (Qiagen, Germantown, MD) overnight at 37ºC at a final concentration of 50 µg/mL then treated with protease inhibitor cocktail (Calbiochem, Billerica, MA) and dialyzed using a 7-kDa molecular weight cutoff microdialysis cassette (Pierce) in PBS. Capsid degradation was confirmed by PageBlue™ staining (Fermentas™, Grand Island, NY) and Western blot analysis.

**Western Blot analysis**

To monitor proteinase K digestion, 5 μg of each protein sample was boiled for 5 min in reducing loading dye (Thermo Scientific, Grand Island, NY), separated on 10% Tris-glycine gels (Pierce), and transferred to nitrocellulose (iBlot® gel transfer stacks, Novex, Grand Island, NY). Membranes were blocked in 5% milk in PBS with 0.1% Tween 20 for a minimum of 30 min and then incubated overnight with 1:500 dilutions of TAstV-2 capsid–specific rabbit polyclonal antibodies against residues R_32_–E_47,_ K_676_–R_691_ (ResGen, Carlsbad, CA), and H_194_–G_211_ (Invitrogen). Antibodies were detected by incubation with HRP-conjugated secondary antibodies (Jackson, West Grove, PA) at a 1:10000 dilution for a minimum of 30 min (Sigma, St Louis, MO) followed by development with SuperSignal^®^ West Dura Extended Duration or Pico Chemiluminescent substrates (Thermo Scientific).

To monitor NHE3 and Na^+^/K^+^ ATPase localization, intestines were collected at 6 hpi and homogenized with a bead beater in 20 mM Tris, 5 mM MgCl_2_, 0.3 mM EGTA, 210 µg/mL sodium fluoride containing protease and phosphatase inhibitors (Pierce). Samples were centrifuged at 11,000 x *g* at 4**°**C to remove debris. Supernatants were collected and incubated for 30 min at 4°C with lysis buffer containing 0.5 % Triton X-100 and centrifuged (12,000 x *g* for 30 min at 4°C). The supernatant containing detergent soluble proteins (predominantly cytosolic proteins) was collected. The pellet was resuspended in lysis buffer containing 0.5% SDS and processed as described above to obtain detergent-insoluble proteins (predominantly membrane proteins). The protein content was quantified by BCA (Pierce), and equivalent amounts were separated on a 10% Tris-glycine gel and transferred to a nitrocellulose membrane before probing with anti-NHE3 (Bioss Antibodies, Woburn, MA, bs-9582R) at a concentration of 2 µg/mL, β-actin (Abcam, Cambridge, MA, ab8227), or Na^+^K^+^-ATPase (Novus Biologicals, Littleton, CO, M7-PB-E9) followed by a secondary HRP-conjugated antibody (Jackson).

**Animal studies**

Fertilized turkey eggs were obtained from a commercial hatchery and incubated at 99.5ºC with humidity greater than 65% until they hatched. Five-day-old poults were randomly assigned to groups (n = 5 – 9 per group) and housed in individual, temperature-controlled Horsfall units with HEPA-filtered inlet and exhaust air valves, where they were given free access to water and routine turkey starter feed. Poults were orally inoculated with 0 to 50 µg purified TAstV-2 capsid in a 200 µL total volume of phosphate-buffered saline (PBS), pH 7.6, 50 µg purified recombinant HAstV-1 capsid protein or PBS alone. Stool from individual birds was scored from 1 to 4 by up to 5 blinded individuals using a scoring system similar to that described by Ball *et al.* ([4](#_ENREF_4)). Scoring was performed at 4 – 6 hpi and then every 12 h thereafter. A score of 3 (liquid or loose stool with some undigested food or solid material) or 4 (watery stool with no solids present) was defined as diarrhea. Scores of at least 3 individuals were combined to generate an unbiased score.

**Immunofluorescent and histological staining**

At the indicated times post-administration, intestinal tissues were embedded in OCT medium (Tissue-Tek, Sakura Finetek, Torrance, CA) and snap-frozen, and sections were prepared at a thickness of 4 µm. The sections were fixed in cold acetone and incubated with antibodies toTAstV2 capsid (K_676_–R_691_ (ResGen, Carlsbad, CA)), as described previously ([5](#_ENREF_5)), SGLT-1 (Millipore, Billerica, MA, 07-1417), claudin 3 (Abcam, ab15102), ZO-1 (Abcam, ab59720), connexin32 (Abcam,ab66613), claudin 1 (Abcam, ab15098), ZO-1 (Invitrogen, ZO1-1A12), and occludin (Invitrogen, OC-3F10). After washing in PBS, the sections were incubated in a mixture of AlexaFluor 488 conjugated secondary antibody (Invitrogen) and phalloidin conjugated with AlexaFluor 555 (for actin staining, Molecular Probes®, Waltham, MA) and 4’,6-Diamidino-2-Phenylindole (DAPI, to stain nucleic acid) (Molecular Probes®). Coverslips were mounted on slides with fluorescent mounting medium (ProLong® Gold, Molecular Probes®), and the sections were examined with a Nikon TE2000 E2 microscope equipped with a Nikon C1Si confocal scanhead. Images were acquired with a Nikon 40x 1.3 NA Plan Fluor objective lens using Nikon EZC1 software using identical parameters between groups. Three-dimensional images were obtained using IMARIS software.

**Astrovirus qRT-PCR assay**

TAstV-2 stocks were prepared from intestines collected from infected turkey poults. Briefly, pieces of intestine were suspended in 0.5 ml PBS in multiple tubes, homogenized using 2-mm zirconium oxide beads (Next Advance, Averill Park, NY) beads for 4 min on speed setting 4 (Next Advance air cooling bullet blender), and pelleted by centrifugation at 12,000 rpm for 5 min. The supernatants were pooled and filtered through a 0.2-μm filter (fecal filtrate), and viral copy number was quantified by real-time RT-PCR. Viral RNA was isolated from homogenates or 10% stool by the QIAamp viral RNA minikit (Qiagen, Germantown, MD) according to the manufacturer's instructions. PCR was performed on 2 µl of each sample using TaqMan^TM^ Fast Virus 1-Step Master Mix (Applied Biosciences, Waltham, MA) with 600 nM forward primer 5′GACTGAAATAAGGTCTGCACAGGT, 600 nM reverse primer 5′AACCTGCGAACCCTGCG, and 200 nM probe 6-carboxyfluorescein (6FAM)-ATGGACCCCCTTTTTCGGCGG-BHQ1 (black hole quencher) under the following conditions: 50°C for 5 min, 95°C for 20 s, followed by 40 cycles, with one cycle consisting of 95°C for 3 s and 60°C for 30 s on a Bio-Rad CFX96 real-time PCR detection system. The number of genome copies/µL of total RNA was determined using a standard curve generated from a synthesized TAstV-2 DNA from nucleotides 4001 to 4201 with a known copy number (calculated using Thermo Fisher Scientific DNA Copy Number and Dilution Calculator). Log_10_ dilutions of the synthesized TAstV-2 DNA were used for real-time RT-PCR as described above.

**Astrovirus capsid ELISA.** ELISA to detect TAstV-2 capsid was performed as previously described ([6](#_ENREF_6)). Briefly, plates were coated with the indicated amount of TAstV-2 capsid or viral homogenates and allowed to incubate overnight. BSA (1 µg/ml) served as a background binding control. Plates were washed 3X with PBS-0.05% Tween-20 (PBST) before blocking with a 5% BSA-PBST solution for 1 hr at room temperature. Plates were washed 3X in PBST and incubated with anti-TAstV-2 antibody (K_676_–R_691_) at a dilution of 1:1000 for 2 h at room temperature. Plates were again washed 3X with PBST and incubated with goat-anti-rabbit conjugated to HRP at a 1:10000 dilution before washing 3X with PBST and developing with substrate (R&D, Minneapolis, MN). The reaction was stopped with 2N H_2_SO_4_ and absorbance was read at 450 nm. Samples were assayed in duplicate.

**Barrier permeability experiments**

Six hours after capsid administration, the ileum was harvested in oxygenated (95% O_2_/5% CO_2_) avian Ringer’s solution ([7](#_ENREF_7)) and mounted in Ussing chambers with 1.3-cm^2^ apertures, as previously described ([8-10](#_ENREF_8)). For each Ussing chamber experiment, tissues from an animal were mounted in duplicate chambers and six animals were used for each experiment. Tissues were bathed on the serosal and mucosal sides with 8 mL oxygenated avian Ringer’s solution circulated in water-jacketed reservoirs. The serosal bathing solution contained 10 mM glucose and was balanced osmotically on the mucosal side with 10 mM mannitol. The spontaneous potential difference (PD) was measured using Ringer-agar bridges connected to calomel electrodes, and the PD was short-circuited through Ag-AgCl electrodes by using a voltage clamp that corrected for fluid resistance to obtain measurements of the short circuit current (*I_sc_*). The transepithelial electrical resistance (TER, Ω•cm^2^) was calculated from the spontaneous potential difference and *I_sc_*, as previously described ([10](#_ENREF_10), [11](#_ENREF_11)). *I_sc_* and PD were recorded at 15-min intervals.

To study paracellular permeability, measurements of mucosal-to-serosal flux of [^3^H]-mannitol were performed at 6 h post-capsid administration by adding [^3^H]-mannitol (0.2 µCi/ml, diluted in 10 mM mannitol) to the mucosal side of Ussing chamber-mounted tissues. After a 15 min equilibration period, samples were taken from the mucosal side of each chamber. A 60-min flux period was established by taking 0.5 mL samples from the serosal compartment opposite the mucosal compartment to which [^3^H] mannitol was added. The presence of [^3^H] was established by measuring β-emission in a liquid scintillation counter (1219 Rack Beta, LKB Wallac, Perkin Elmer, Waltham, MA).

**Statistics**

Data were analyzed by Student’s *t-*test (TER and paracellular permeability) or one-way ANOVA (diarrhea scores and NHE3 localization) using GraphPad Prism v6 software (La Jolla, California). *p*-values < 0.05 were considered significant.

**Supplemental Figure Legends**

**Supplemental Fig. 1. TAstV-2 infection in turkey poults.** (A) Clinical scoring chart to assign diarrhea scores. (B) Turkey poults (n = 4 – 6 per group) were orally inoculated with 1.5 x 10^13^ genome copies of TAstV-2 virus and were monitored for clinical score (B) reported as the percent of animals with diarrhea (score of 3 or higher) over time. Viral copy number was monitored on stool (C) and intestinal homogenates (D) by quantitative real time RT-PCR at the indicated times.

**Supplemental Fig. 2. Quantification of TAstV-2 virus.** (A) ELISA was performed on TAstV-2 capsid (25 ng) serially diluted 2-fold in PBS or intestinal homogenate from uninfected poults (control), viral homogenate, or control homogenate alone.

**Supplemental Fig. 3. Proteinase K digestion of TAstV-2 capsid.** (A) PageBlue^™^ protein staining of whole and proteinase K–digested TAstV-2 capsid (50 µg). (B) Purified TAstV-2 capsid was incubated with proteinase K overnight at 37ºC at a final concentration of 50 µg/mL then treated with protease inhibitor cocktail and dialyzed using a 7-kDa molecular weight cutoff microdialysis cassette in PBS. Capsid degradation was confirmed by immunoblot using anti-TAstV-2 capsid antibodies.

**Supplemental Fig. 4. TAstV-2 capsid inoculation does not cause intestinal damage or inflammation.** Intestinal sections collected from turkey poults inoculated orally with PBS (control) or 50 μg recombinant TAstV-2 capsid protein (capsid) were collected at 6 hpi and stained with hemotoxylin and eosin. Scale bar = 25 µm.

**Supplemental Fig. 5. Immunofluorescent staining of turkey intestines for tight junction proteins.** Uninfected mice or turkey poults were sacrificed and intestinal sections were stained for tight junction proteins (green) and nuclei (blue). Scale bar = 20 µm.

**Supplemental References**

1. **DuBois RM, Freiden P, Marvin S, Reddivari M, Heath RJ, White SW, Schultz-Cherry S.** 2013. Crystal structure of the avian astrovirus capsid spike. J Virol **87:**7853-7863.

2. **Koci MD, Kelley LA, Larsen D, Schultz-Cherry S.** 2004. Astrovirus-induced synthesis of nitric oxide contributes to virus control during infection. J Virol **78:**1564-1574.

3. **Moser LA, Carter M, Schultz-Cherry S.** 2007. Astrovirus increases epithelial barrier permeability independently of viral replication. J Virol **81:**11937-11945.

4. **Ball JM, Tian P, Zeng CQ, Morris AP, Estes MK.** 1996. Age-dependent diarrhea induced by a rotaviral nonstructural glycoprotein. Science **272:**101-104.

5. **Nighot PK, Moeser A, Ali RA, Blikslager AT, Koci MD.** 2010. Astrovirus infection induces sodium malabsorption and redistributes sodium hydrogen exchanger expression. Virology **401:**146-154.

6. **Meliopoulos VA, Kayali G, Burnham A, Oshansky CM, Thomas PG, Gray GC, Beck MA, Schultz-Cherry S.** 2014. Detection of antibodies against Turkey astrovirus in humans. PLoS One **9:**e96934.

7. **Whitsel AI, Johnson CB, Forehand CJ.** 2002. An in ovo chicken model to study the systemic and localized teratogenic effects of valproic acid. Teratology **66:**153-163.

8. **Argenzio RA, Liacos JA.** 1990. Endogenous prostanoids control ion transport across neonatal porcine ileum in vitro. Am J Vet Res **51:**747-751.

9. **Argenzio RA, Liacos JA, Levy ML, Meuten DJ, Lecce JG, Powell DW.** 1990. Villous atrophy, crypt hyperplasia, cellular infiltration, and impaired glucose-Na absorption in enteric cryptosporidiosis of pigs. Gastroenterology **98:**1129-1140.

10. **Little D, Dean RA, Young KM, McKane SA, Martin LD, Jones SL, Blikslager AT.** 2003. PI3K signaling is required for prostaglandin-induced mucosal recovery in ischemia-injured porcine ileum. Am J Physiol Gastrointest Liver Physiol **284:**G46-56.

11. **Blikslager AT, Roberts MC, Argenzio RA.** 1999. Prostaglandin-induced recovery of barrier function in porcine ileum is triggered by chloride secretion. Am J Physiol **276:**G28-36.
